# An Integrated Data Acquisition Approach for the Structural Health Monitoring and Real-Time Earthquake Response Assessment of a Retrofitted Adobe Church in Peru

**DOI:** 10.3390/s24165327

**Published:** 2024-08-17

**Authors:** Georgios Karanikoloudis, Alberto Barontini, Nuno Mendes, Paulo B. Lourenço

**Affiliations:** Department of Civil Engineering, University of Minho, 4800-058 Guimarães, Portugal; albertobarontini@civil.uminho.pt (A.B.); nunomendes@civil.uminho.pt (N.M.); pbl@civil.uminho.pt (P.B.L.)

**Keywords:** structural health monitoring, condition assessment, dynamic identification tests, out-of-plane rotations, crack propagation, traditional retrofitting techniques, adobe heritage structures, large data series processing

## Abstract

The structural health monitoring (SHM) of buildings provides relevant data for the evaluation of the structural behavior over time, the efficiency of maintenance, strengthening, and post-earthquake conditions. This paper presents the design and implementation of a continuous SHM system based on dynamic properties, base accelerations, crack widths, out-of-plane rotations, and environmental data for the retrofitted church of Kuñotambo, a 17th century adobe structure, located in the Peruvian Andes. The system produces continuous hourly records. The organization, data collection, and processing of the SHM system follows different approaches and stages, concluding with the assessment of the structural and environmental conditions over time compared to predefined thresholds. The SHM system was implemented in May 2022 and is part of the Seismic Retrofitting Project of the Getty Conservation Institute. The initial results from the first twelve months of monitoring revealed seasonal fluctuations in crack widths, out-of-plane rotations, and natural frequencies, influenced by hygrothermal cycles, and an apparent positive trend, but more data are needed to justify the nature of these actions. This study emphasizes the necessity for extended data collection to establish robust correlations and refine monitoring strategies, aiming to enhance the longevity and safety of historic adobe structures under seismic risk.

## 1. Introduction

Earthen buildings are prone to early structural damage, including cracking, the separation of structural elements, and often the collapse of large sections, due to their low mechanical properties, lack of connections between structural parts, brittle behavior, and critical out-of-plane resistance when subjected to seismic actions [1,2]. Additionally, factors such as poor maintenance, harsh environmental conditions characterized by wet–dry cycles—which are likely to intensify due to climate change—and the absence of adequate retrofitting techniques further contribute to the degradation of these historic structures.

The structural condition of an existing building and the performance of any interventions implemented during its service life need to be assessed and monitored over time to detect damage and track its evolution. The primary goals are early damage detection, efficient maintenance planning, and ensuring safety. Structural health monitoring (SHM) methods enable the continuous, long-term observation of structural conditions and actions. In recent years, SHM has gained significant attention for its potential to enhance the safety and longevity of historic structures, as demonstrated in [3,4,5,6,7]. Active phenomena related to structural deformations and cracking are often difficult to interpret, in terms of severity, magnitude, and time evolution, even using the most advanced numerical analyses, unless data are obtained from permanent instrumentation and for periods covering several years. Also, both structural and environmental parameters must be recorded and correlated with causes and effects. Thus, an SHM plan for a historic structure, together with periodic inspections and a maintenance plan, are among the most efficient tools for seismic risk mitigation and for securing the integrity of historic properties, especially under the philosophy of minimal intervention. As shown in Sivori et al. [8], the continuous application of the SHM of the dynamic properties of historic structures at seismic zones can provide further attributes regarding the concept of a digital twin (DT), calibrating numerical models over time, and evaluating the capacity after each seismic event. In Kita et al. [5], the continuous monitoring of natural frequencies and crack widths of the Consoli Palace in Gubbio, Italy, revealed a negative linear or second-order polynomial and a positive linear correlation with the ambient temperature, respectively. On the contrary, in Cavalagli et al. [4], the SHM of the belltower of the Basilica of San Pietro in Perugia revealed a positive linear correlation between the ambient temperature and the two out-of-plane modes, while a negative one was observed for the tortional mode. Accounting also for the dynamic data to be used for damage detection, a series of nonlinear dynamic analyses generated damage-induced curves for normalized modes. Accounting for the SHM of static data, i.e., out-of-plane rotations and crack widths, time evolution was analyzed as being a combination of a linear trend, following static actions, and a sinusoidal trend, the result of seasonal variations. Finally, in Gentile et al. [7], SHM data collection for 15 months from the Gabbia masonry tower in Mantua, Italy revealed the positive linear regression of the natural frequencies and ambient temperature.

Interpreting active phenomena related to structural deformations and cracking—in terms of severity, magnitude, and time evolution—can be challenging, even with advanced numerical analyses, unless data are obtained from permanent instrumentation over several years. Additionally, both structural and environmental parameters must be recorded and correlated with their causes and effects. Therefore, implementing an SHM plan for a historic structure, along with periodic inspections and a maintenance plan, are among the most effective tools for mitigating seismic risk and ensuring the integrity of historic properties, particularly under the philosophy of minimal interventions. As presented in Borlenghi et al. [9], a training period covering 6 to 12 months of monitoring data is sufficient to establish a linear regression between environmental and static or dynamic data. Yet, in the case of highly deformable earthen structures, correlations under extreme seasonal environmental parameters and static or dynamic data are often nonlinear, and longer training periods of SHM data collection are needed to achieve damage identification.

The church of Santiago Apóstol de Kuñotambo, located in Acomayo, Cusco, Peru, is a 17th-century religious structure (1681) representative of the churches built in the Andes during the Spanish Viceroyalty period. As a typical example of adobe churches in the Altiplano, it features a single nave leading to an elevated presbytery and altar, with an adjacent sacristy and baptistery, along with a belltower. The walls and buttresses are constructed of adobe masonry, set on a plinth of rubble stone masonry with earthen mortar, and topped with a single gable timber roof.

Under the ongoing Seismic Retrofitting Project by the Getty Conservation Institute (GCI), the church serves as a field laboratory for the assessment and conservation of its structural elements and wall paintings. This project emphasizes the use of traditional and locally available materials and techniques, ensuring alignment with the church’s historical context and integrity.

The structural integrity of the church was assessed in various stages of its service life, divided into five main phases:

1. Initial assessment (May 2015): The work began with in situ inspections and non-destructive testing, including ambient vibration testing (AVT) and sonic testing. This was followed by numerical modeling and structural analysis to replicate the existing damage and evaluate the current safety through nonlinear static and dynamic analyses [2,10].
2. Retrofitting scheme development (2016): A retrofitting scheme using traditional and locally available materials and techniques was developed, considering the presence of wall paintings on the interior surfaces. The University of Minho (UMinho) numerically validated the retrofitting design and provided optimization suggestions, incorporated in the final design. The strengthening scheme primarily involved internal and external bracing with timber elements, such as bond beams, tie beams, anchors, and corner keys, as well as the addition of buttresses (presented in detail in [2,11,12]). It also included the consolidation and partial replacement of damaged exterior adobe and base course masonry parts.
3. Retrofitting completion (June 2019): The retrofitting works were completed by the Cusco Directorate of the Ministry of Culture of Peru, culminating in the church’s inauguration and official reopening.
4. Post-retrofit assessment (December 2019, 2022): UMinho conducted a second in situ inspection and experimental round of AVT and sonic testing in December 2019. In May 2022, additional damage mapping of the retrofitted church was conducted based on the definition of main structural damage in earthen buildings, presented in Ahmadi et al. [13], which documented the formation of cracking, indicative of active destabilizing actions, possibly settlement.
5. Long-term monitoring (May 2022): The installation of a permanent long-term SHM system by UMinho records structural, environmental, and earthquake parameters.

The main contribution of the current work is to present the methodological approach involving the inspection and diagnosis of the church of Santiago Apóstol de Kuñotambo, the design and installation of SHM devices for its condition monitoring, and the analysis of initial results. The sequence of inspections and non-destructive testing (NDT) allowed for the optimization of the monitoring system and the comparison of the initial findings. Here, the state of retrofitting adds additional complexity in the assessment, common in highly deformable adobe structures. Hence, besides the enhancement in lateral capacity, the structure enters an adjustment phase due to deformations from drying shrinkage in new adobe structural elements, cyclic expansions and contractions of embedded timber elements, and the additional load from the new roof.

The installation of the SHM system in the church of Santiago Apóstol de Kuñotambo was carried out in May 2022, encompassing data such as natural frequencies and modes of vibration, out-of-plane tilting, and cracks, correlated with temperature, relative humidity, and strong motion seismic data. Here, the results of the first 12 months of data collection are presented and discussed.

## 2. Equipment and Methods

The permanent SHM system for the church of Kuñotambo was designed based on prior knowledge of structural conditions, field inspections, diagnostic campaigns, and structural assessment processes. The system provides continuous hourly observations of parameters relevant to structural performance, as defined in [14]. First, the main focus is on monitoring structural conditions and destabilizing actions by recording changes in out-of-plane tilting and crack opening, correlated with environmental data, i.e., temperature and relative humidity, as well as seismic data. Here, temperature and relative humidity will help to establish seasonal trends, while seismic data from the base of the church will establish the nature and amplitude of seismic events. Second, the dynamic properties of the structure, i.e., natural frequencies and mode shapes, are monitored based on hourly ambient vibration acquisitions. Their continuous assessment over time has been proven to be indicative of damage detection, as stated in [3,4,5,6,7,15], and constitutes an efficient tool for structural assessment over time, with a focus on post-earthquake response, i.e., seismic structural health monitoring (SSHM). Dynamic data also present seasonal and daily fluctuations, following temperature and relative humidity, and trends should be established after the first seasonal cycles.

**Figure 1:**
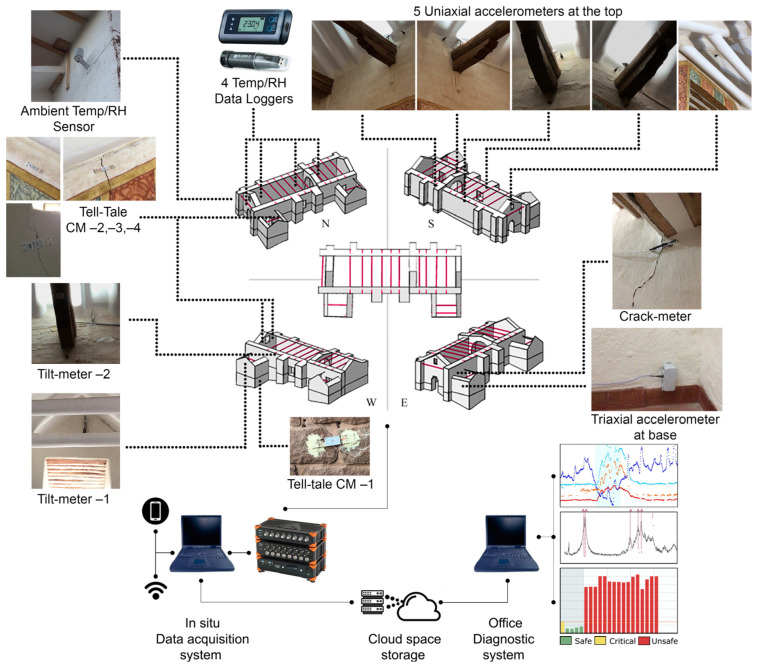
Schematics of a long-term SHM plan for the church of Kuñotambo. Dashed lines point the locations of sensors and dotted lines connect the components and processes of the SHM system.

Cost–benefit criteria were also applied to the design of the system. Instrumentation was carefully selected in terms of sensitivity and environmental exposure and tailored to the specific objectives in order to select the most efficient SHM devices. The SHM system recorded continuously in acquisitions of 60 min. As shown in Figure 1, the SHM system consisted of:

(a) One exterior air temperature and relative humidity sensor. The sensor is located at the top east end of the south lateral wall. It is protected by solar radiation and precipitation shield. The sampling rate for both sensors is set to 20 Hz (20 samples/s). The measurement range is 0–100% RH and −40–+80 °C, with a protection of IP65 and a measurement accuracy of ±1.5% RH (0–90% RH) and ±0.2 ° (0–+40 °C). Model: HUMICAP Probe HMP110. Manufacturer: Vaisala (Vantaa, Finland).
(b) Five accelerometers at the top eaves of the nave and presbytery walls. The sensors are piezoelectric uniaxial accelerometers with a frequency range of 0.15 to 1000 Hz, a measurement range of ±0.5 g, and a high sensitivity of 10,000 mV/g. The signals are recorded with a sampling frequency equal to 200 Hz (200 samples/s). The sensors are mounted with small anchors on the interior surfaces: two at the top eave of the north nave and presbytery wall, two in the south nave and presbytery wall, and one at the top of the gable end of the east façade. Model: 393B12. Manufacturer: PCB Piezotronics (Depew, NY, USA).
(c) One linear potentiometer crack-meter, anchored to the adobe wall of the staircase leading to the choir loft, crossing a large vertical crack, which was present before the retrofitting works. The sensor has a measuring range of ±25 mm, an accuracy of ±0.0625 mm, and a protection of IP67. It is installed with a PT100 temperature sensor (Manufacturer: Rs-components, London, UK) on the side, embedded in the adobe wall. The sampling rate is set to 20 Hz (20 samples/s). Model: J6-1-25. Manufacturer: Soil Instruments (Uckfield, UK).
(d) Two MEMS tilt-meter sensors are placed on the north nave wall and gable end of the baptistery to monitor vertical rotations. Their angle-measuring range is ±5° and their accuracy is ±0.05%. They are installed with a PT100 temperature sensor (Manufacturer: Rs-components, London, UK), one on each side, embedded in the adobe wall. The sampling rate is set to 20 Hz (20 samples/s). Model: TLT6-U-5 MEMS. Manufacturer: Soil Instruments (Uckfield, UK).
(e) One triaxial accelerometer, mounted on the interior surface, at the base of the east side wall of the baptistery to record strong motions from seismic events. It has a high resolution (0.0008 m/s²), a sensitivity of 100 mV/g, a measurement range of ±50 g pk, and a frequency range of 0.5 to 5000 Hz (±5%). The signals are recorded with a sampling frequency equal to 200 Hz (200 samples/s). Model: 356A16. Manufacturer: PCB Piezotronics (Depew, NY, USA).
(f) One data acquisition system of two versatile 24-bit USB data acquisition boards with sixteen channels (eight IEPE channels and eight voltage channels). Acquisition recordings are simultaneous for all sensors, considering different specifications and sampling rates. Both acquisition boards are connected to a laptop, two uninterruptible power supply (UPS) units, offering about 1 h of power autonomy during an electricity cutoff, and a Wi-Fi router. Model: SIRIUS. Manufacturer: Dewesoft (Trbovlje, Slovenia).

In parallel, and not part of the main SHM system, additional data were collected through four tell-tale crack-meters, following the evolution of three major cracks, i.e., at the gable end of the east façade and the west side wall of the baptistery. In addition, four air temperature and relative humidity data loggers were placed in selected locations: one in the exterior balcony of the choir loft and three in locations of presbytery, nave, and choir loft. Their distribution served to identify potential environmental variations within the interior of the church, while placed in non-visible recesses of niches, timber pieces, and the choir loft balcony.

An important aspect in SHM is the framework of post-processing and the assessment of the evolutionary structural and environmental conditions. Here, given the remote location of the church and the limited internet connection, the collection and organization of data—dynamic, static, and environmental—was performed twice per year, in the scheduled field campaigns during April–May and September–October. Next, first-level signal processing was applied to the data, involving basic checks on the quality of the signals. In the case of noise in the dynamic data, basic data treatment techniques were applied, i.e., removal of constant or average (DC) components, digital filtering in frequency domain, etc. Yet, filtering should target the noise frequency range and always be limited, as it can mask real data profiles, especially in wide range noise frequency bandwidths [16]. In the current SHM application, the infrequent use of the church, with mass and religious activities limited to once or twice per month, and the choice of instrumentation and acquisition specifics—sensitivity, sampling ratios, etc.—ensured a high signal-to-noise ratio (SNR) and thus, no further cleaning or filtering of data was necessary. Next, down-sampling was performed for static and environmental data, from 20 samples/s to hourly median values, while the acceleration data of 200 samples/s were kept intact. Here, it was noted that the median value of a dataset was more resistant to extreme values and outliers. In fact, in the presence of skewed data, the median provided a better representation of the center compared to the mean value, which is often the case for environmental and static data containing seasonal and daily variations. Following, the large number of time series, processing was performed automatically with scripts to extract basic statistical values: mean and median values, distribution and variability, histograms, etc. For the ambient vibration data, automated operational modal analysis (OMA) scripts extracted frequencies and mode shapes over time. In parallel, a range of threshold values and damage indicators should be defined for each of the monitoring parameters, accounting for numerical analyses, performance limit states, and the literature, as described by Cattari et al. [17]. Finally, after the collection of at least two seasonal cycles, the assessment of monitoring data was performed by regression analyses of structural and environmental parameters, establishing evolution trends and applying time predictions [18]. Hence, rigorous checks of threshold safety levels can be maintained for all monitored structural parameters, and through future projections, early warnings can be issued for informed decision making.

## 3. Results

The SHM system was installed in the church on 7 May 2022, and has been recording hourly acquisitions ever since. During this period, a few downtimes occurred due to power failures, larger in duration than the autonomy of the UPS units. The system allows the users to remotely verify that it is functioning correctly; thus, upon a downtime occurrence, a warning is sent onsite, and the operation is re-established manually, often creating gaps in time series. Hereafter, the SHM data collected between 7 May 2022 and 26 May 2023 are discussed.

### 3.1. Ground Motion Recording

**Figure 2:**
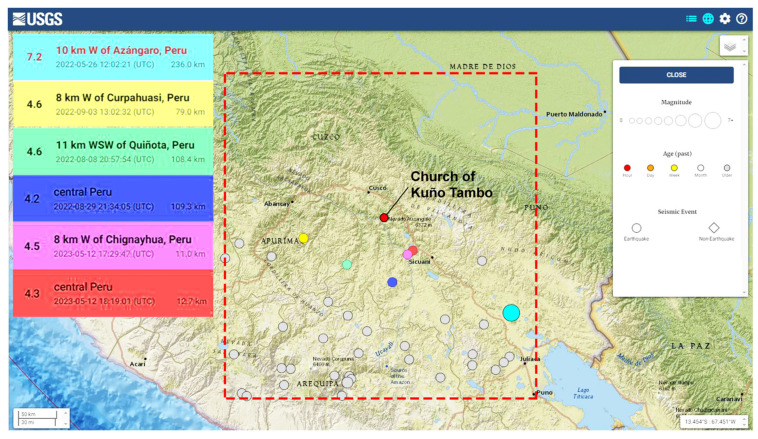
Geographic location of the church of Kuñotambo (red dot) and locations of earthquakes with M > 4.0 within the area of 300 km^2^ contained in the red dashed square, from May 2022 to May 2023 [22]. Note the earthquake sources near the church in different colors.

**Figure 3:**
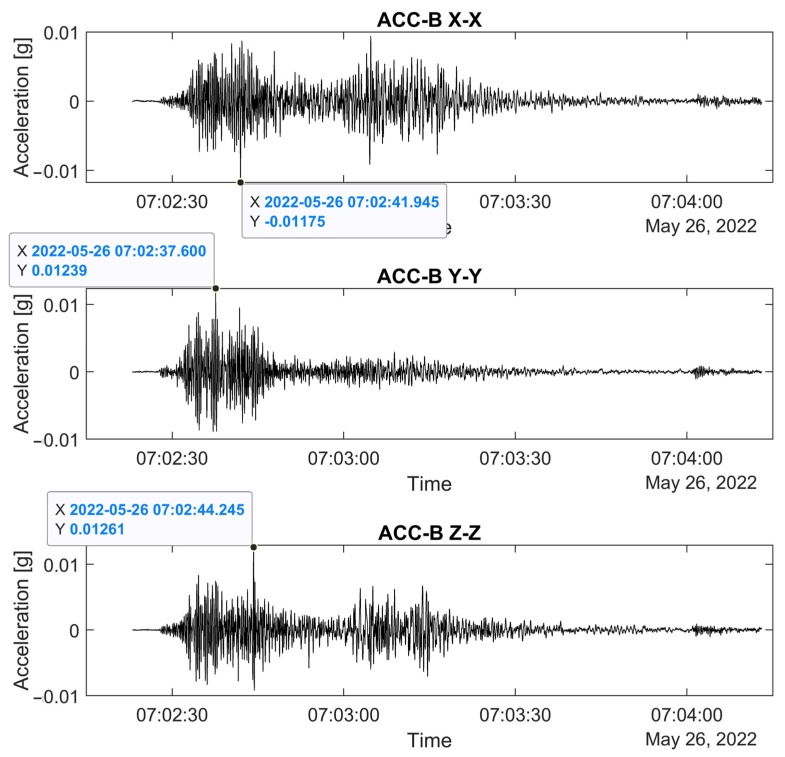
Cropped acceleration–time histories at the base during the recorded earthquake event (26 May 2022, 7:02 a.m. local Peruvian time).

**Figure 4:**
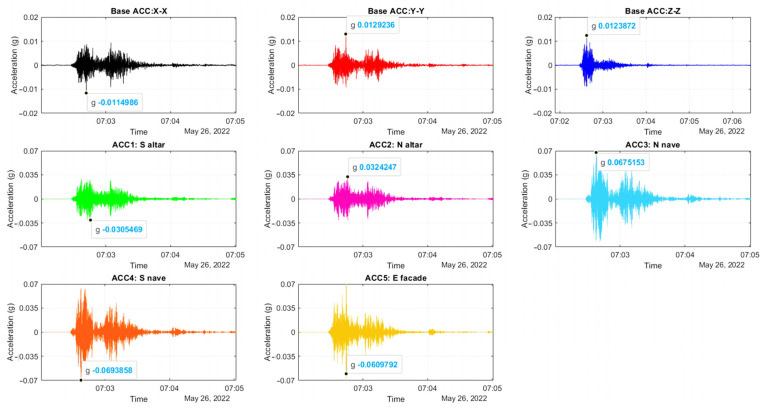
Cropped acceleration–time histories at the top of the walls (all in horizontal direction) during the recorded earthquake event on 26 May 2022, at 7:02 a.m. local Peruvian time.

The triaxial accelerometer at the base continuously records in order to capture motion due to seismic events. The processing uses automatic phase picking to detect the arrival time of an earthquake and then to isolate and store the record in three directions. The automatic peak picking is based on a short time average (STA) and a long time average (LTA) threshold method, which is a well-established and widely used method, presented in Allen [19], based on the statistical properties of the earthquake signal. This consists of the comparison of the ratio between two moving average windows, STA and LTA (STA/LTA), applied to the absolute value of the detrended acceleration signal. The STA window is related to the seismic event and its dominant frequency, with small windows corresponding to more local events of shorter durations. The LTA is sensitive to the noise level of the signal. Here, the STA and LTA windows are optimized through trials and set equal to 0.2 s and 100 s, respectively. The choice of a threshold for the STA/LTA ratio depends on the SNS levels in the signals, the range of earthquake amplitudes, and the size of STA and LTA windows, e.g., equal to 50 in [20] and 10 in [21]. In the current study, instead of a fixed value for the STA/LTA ratio for the earthquake event detector, a threshold condition is assigned and calculated for each signal, which is the ratio of a reference acceleration of 0.001 g (1 mg) and the median absolute value of the signal, around 0.1 mg, the latter corresponding to the level of base acceleration with no excitation. Once the STA/LTA ratio passes the assigned threshold, a probable earthquake event is detected, pre-trigger and post-trigger time increments of 10 s and 100 s are added, and the event is cropped and stored. A second-level selection follows, involving a manual check of the cropped signals to identify true earthquake signals and discard those related to noise and other sources. Finally, selected earthquake signals are cross-referenced with earthquake databases. Here, to establish an earthquake catalogue relevant to the structure, an area of influence is chosen, defined by a rectangle with a diagonal of around 570 km, and centered on the structure (Figure 2). From May 2022 to May 2023, out of a total of 37 events with a magnitude M > 4.0, the 5 most significant earthquakes that occurred near the structure (58–108 km) are shown in Figure 2. In addition, the earthquake with the highest intensity in the area is included, which occurred on 26 May 2022, at 7:02 a.m. local Peruvian time, with a magnitude of M7.2 and an epicenter near Puno, around 200 km from the structure. This earthquake is the only one selected through the applied detection method. The time of arrival of the first earthquake wave is approximately 20 s after its generation. The plots for the base triaxial accelerometer sensor have very similar amplitudes in the three directions (X–X: horizontal N–S; Y–Y: vertical; Z–Z: horizontal E–W), with a maximum horizontal acceleration of 0.012 g (Figure 3). The plots of the horizontal accelerometers at the top of the walls show an amplification in elevation, up to 7 times, with the highest amplitude of 0.069 g observed in the nave walls and 0.061 g at the gable end of the east façade (Figure 4). A second wave was captured in all directions, though of lower amplitude in the vertical direction (Z–Z) (Figure 3 and Figure 4).

**Figure 5:**
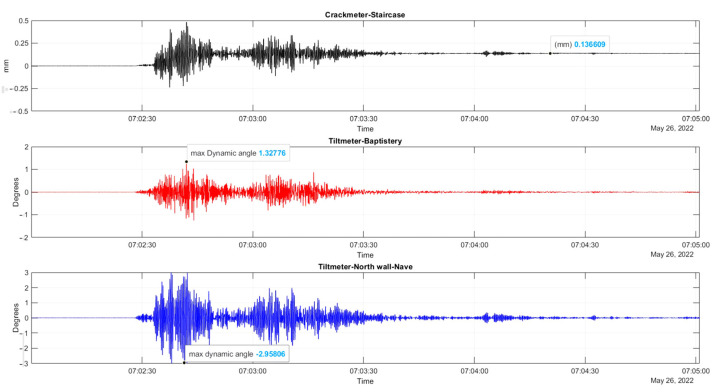
Cropped evolution graphs of the crack-meter and two tilt-meters during the earthquake on 26 May 2022, at 7:02 a.m. local Peruvian time.

The plots of the crack-meter and tilt-meters, as shown in Figure 5, also measure the earthquake effects on the walls, in mm and degrees, respectively. From the plot of the crack-meter, a permanent small opening in the crack of the staircase void is recorded (around 0.14 mm). The tilt-meters measure increased dynamic angles during the earthquake, but no permanent rotation is recorded, as also observed in other SHM applications [9].

### 3.2. Environmental Parameters

The weather in Kuñotambo, part of the Peruvian altiplano region, is characterized by two fairly distinct seasons, i.e., a dry season during May–October, in which the average monthly precipitation falls below 50 mm, and a wet–rainy season during November–April, in which 88% of the annual precipitation occurs [23]. The external air temperature (Temp) and relative humidity (RH) data are recorded via an ambient Temp/RH PT100 sensor, placed in the exterior southeast front of the church. Considering the internal temperature, three PT100 temperature sensors are coupled with the static sensors, i.e., the crack-meter (CR) and the two tilt-meters, namely T1 at the gable end of the baptistery and T2 on the top eave of the north nave lateral wall. For all the above-mentioned sensors, acquisition recordings are configured with a sampling frequency of 20 Hz. In the interior of the church, the internal air temperature and RH are also continuously monitored using three independent dataloggers, located in different locations of the nave.

**Figure 6:**
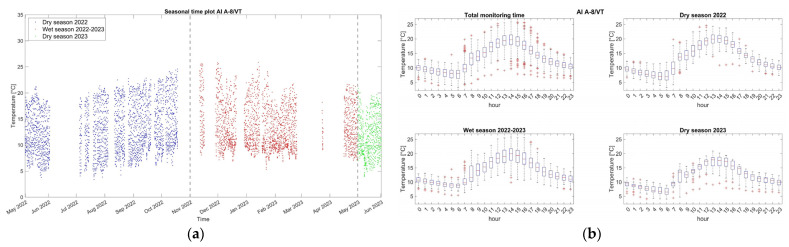

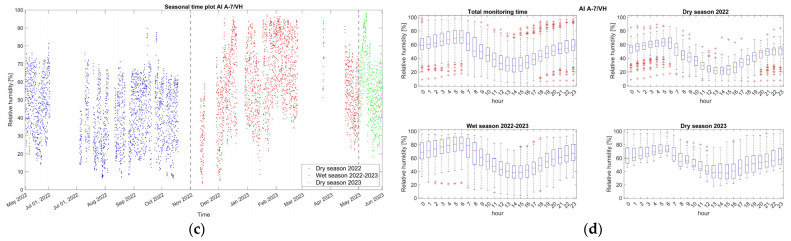
Ambient Temp/RH PT100 sensor: Hourly evolution graphs and boxplots of the hourly datasets for external air temperature (**a**,**b**) and external ambient relative humidity (**c**,**d**) during the entire monitoring period. Note the seasonal divisions in different colors.

**Figure 7:**
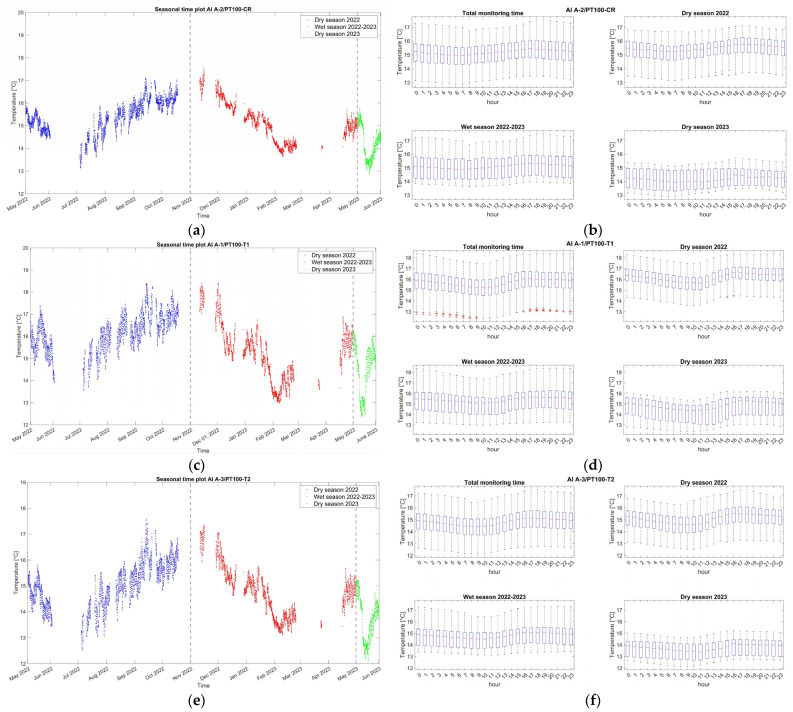
Hourly evolution graphs of interior wall temperatures of PT100 temperature sensors and hourly boxplots (**a**,**b**) close to the crack-meter, (**c**,**d**) close to tilt-meter 1, and (**e**,**f**) close to tilt-meter 2, respectively. Note the seasonal divisions in different colors.

Environmental data, originally sampled at 20 Hz, are first processed to obtain single hourly data, equal to the signal median value. With respect to other statistics, the median is preferred here due to being less sensitive to extreme values and outliers, especially in skewed data. The main statistics of the single hourly data evolution during the monitoring period are presented in Table 1, Table 2 and Table 3. Time plots of the hourly evolution of external air temperature and relative humidity during the monitoring period are presented in Figure 6a,c and Figure 7a,c,e. The statistical variation in the datasets over the monitoring period is shown in the boxplots of Figure 6b,d and Figure 7b,d,f.

From the time evolution plots of Figure 6, clear daily and seasonal fluctuations emerge for the environmental parameters. The external air temperature increases during the first dry season (May–October 2022) and then decreases during the following wet season (November 2022–April 2023). The external RH follows the reverse daily and seasonal evolution. However, this fluctuation couples with an upshift trend during the wet season, characterized by higher values and an increase in the median value from 43% (dry season) to 60% (wet season). Additional peaks, mostly in the external RH, e.g., at the beginning of the second dry season (May 2023), are likely the result of extreme precipitation. In terms of daily fluctuations (boxplots of Figure 6), the median of the external air temperature decreases exponentially from 2 p.m. (highest temperature) until 6 a.m. and increases parabolically from 6 a.m. until 2 p.m., while the external RH increases from 2 p.m. until 6 a.m. (highest RH) and then decreases, in both seasons.

**Figure 8:**
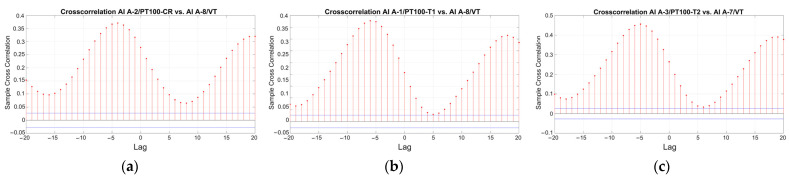
Cross correlation graphs: (**a**) the wall temperature near the crack-meter and the external ambient air temperature, (**b**) the wall temperature near tilt-meter 1 and the external ambient air temperature, and (**c**) the wall temperature near tilt-meter 2 and the external ambient air temperature. Note the time lag in hours.

Regarding the wall temperature, the three sensors located on the internal faces present almost identical values (Figure 7a,c,e), with the median varying between 14.8 °C and 15.7 °C. The range of variation in these records due to seasonal fluctuation is much less than the external, with extreme minimum and maximum values equal to 12.1 °C and 18.4 °C (Table 1). Considering the daily variation (boxplots of Figure 7b,d,f), in both seasons, the median of the wall temperature in the measured three locations exhibits an increase from 9 a.m. until around 5 p.m., followed by a decrease from 5 p.m. to 9 a.m. As also shown in the cross-correlation graphs of Figure 8, the time lag between the internal wall temperature and the external air temperature during the monitoring period is measured around 4–6 h, a range that also reflects the thickness, thermal mass, and location of the corresponding adobe walls. Here, it is noted that once time lags are precisely identified and removed, the correlation between structural and environmental data can be improved.

Finally, the interior environmental parameters in the church, namely internal air temperature and RH, vary within the range of 10.5 °C to 18.8 °C, and 15% to 77%, respectively, with median values equal to 14 °C and about 50%, respectively (Table 1). Seasonal variations are also present in the interior, although they only slightly affect the temperature. The highest differences are identified in the internal RH values, with the median, minimum, and maximum values during the wet season, increasing by 1.36, 1.13, and 1.17 times the respective values during the dry season.

Compared to the exterior, the median internal air temperature is found to increase by 1.15 times, while the median internal RH is almost the same (Table 1). Yet, the maximum values of internal RH are found to be reduced by 0.74 times. In fact, even if there is no mechanical system to control the internal temperature and humidity, the internal environmental conditions are more stable and present less variation compared to the exterior, which is attributed to the insulation ability of the thick adobe walls and the fact that during most days the church remains closed.

### 3.3. Crack Opening

**Figure 9:**
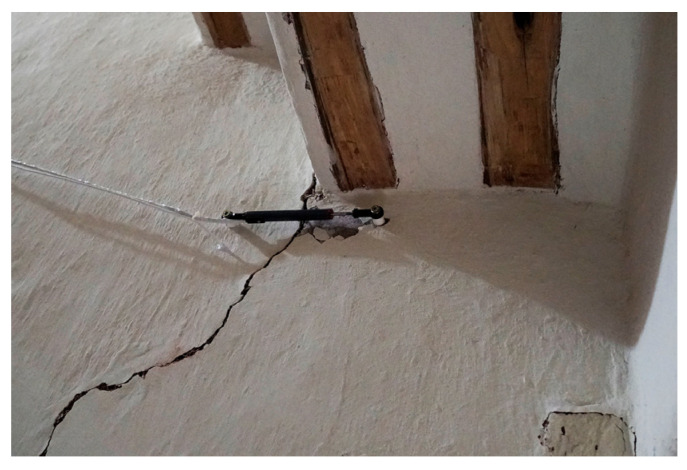
View of the LVDT crack-meter and the adjacent PT100 thermocouple monitoring the crack evolution and wall temperature in the staircase void.

As previously described, a linear potentiometer electromechanical sensor is mounted in the vertical crack at the staircase void. In addition, a PT100 thermocouple, inserted in the wall nearby, records the temperature (Figure 9). The sensors are synchronized with a sampling frequency of 20 Hz (20 samples/s). Since the data refer to a quasi-static condition of the structure, down-sampling is performed to 1 sample/h, with the median value per hour as the timestamp, which provides a better representation of the center of the dataset. As for the environmental parameters, the records of the crack-meter (Figure 9), originally at 20 samples per second, are first processed to obtain a single hourly value equal to the median of the signal. Here, it is noted that the crack pattern in the staircase void is part of the structural damage of the baptistery and the northeast front of the nave, which is either dormant or active, and associated with the sliding of the foundation and/or substrate of the baptistery and/or with excessive thrust from the roof of the nave.

**Figure 10:**
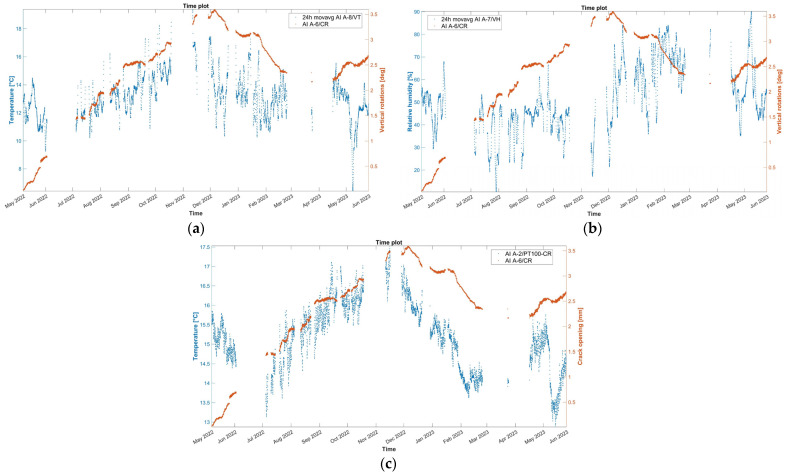
Hourly evolution graphs of crack width and (**a**) 24 h moving average of the external air temperature, (**b**) 24 h moving average of the external RH, and (**c**) wall temperature.

The time plot of the hourly evolution of the crack opening is presented in Figure 10, together with the 24 h moving average of the external air temperature, external RH, and the internal wall temperature at the same location of the crack-meter. The crack opening records the variation from the initial value, set to zero in the graphs, and equal to the initial crack width, namely 6 mm, as measured in May 2022 before installing the sensor. During the first dry season (May–October 2022), a steady increase in the width is noted, with a rate of around 0.6 mm per month, reaching a maximum of 3.6 mm in December 2022. Next, in the first wet season (November 2022–May 2023), the correlation with time becomes negative at a rate of 0.4 mm per month. In these two seasons, the crack opening presents a high positive correlation with both the internal and external wall temperature. Approaching the end of the wet season (April 2023), the correlation with time reverses and the crack opens again. During the dry season, adobe masonry (and the foundation soil) is expected to shrink, causing the opening of cracks, while in the rainy season, the expansion of adobe masonry (and possibly the foundation soil) is causing the cracks to close, also identified in [24]. Yet, the crack opening during the first dry season does not totally recover, which is indicative of a permanent deformation, and a new state of cyclic oscillation is initiated around +2.75 mm with respect to the initial crack width.

Accounting also for the effect of seismic events, as seen in the closeup of Figure 5, during the far distant earthquake recorded (26 May 2022, 7:02 a.m. local Peruvian time, M7.2, 10 km of Azángaro, Peru), with an epicenter of 300 km from the church, a small permanent jump of 0.14 mm in the crack width is recorded. The current crack opening increment is considered to be of minor importance and appears not to influence the time trend evolution of crack width, yet it demonstrates the substantial effect of earthquake actions in structural conditions.

**Figure 11:**
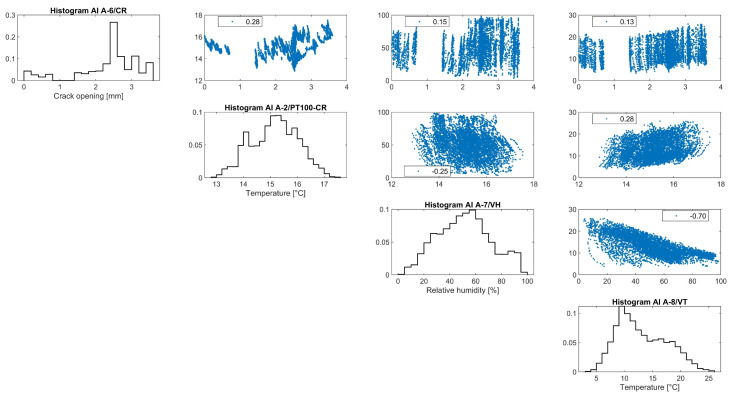
Histograms and correlation graphs of crack width, the corresponding wall temperature, the external air temperature, and RH of the entire monitoring period, in the form of a matrix.

**Figure 12:**
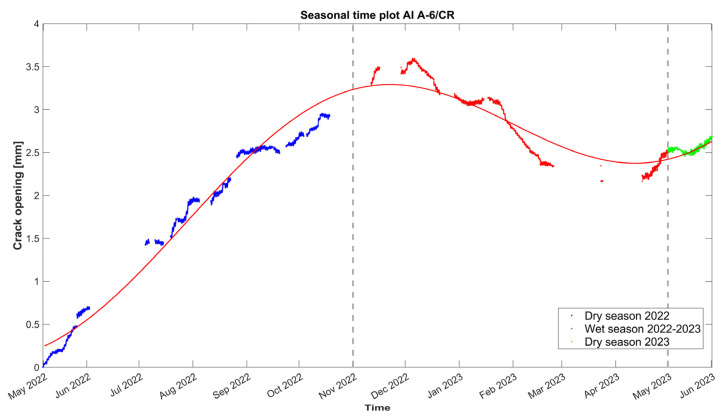
Hourly evolution graph of the crack width, with a nonlinear fit in red. Note the seasonal divisions in different colors and the *x*-axis in months. The dashed lines signify the seasonal divisions.

Considering the correlation graphs of Figure 11, the linear regression between the crack opening and the environmental parameters appears poor, which can be mostly associated with time lags due to the thermal inertia of the adobe. According to [18], a simple nonlinear regression is fitted, in Figure 12, to the time evolution *t* of the crack opening *CR* data by considering the combination of a linear equation *Bt + C*, with a slope *B* that accounts for the evolution rate of the residual crack width and a y-intercept *C*, and a sinusoidal equation *Asin (Pt – φ)*, to account for what is assumed to be a seasonal cycle. Here, *A* is the amplitude of the signal, defined as the absolute average of the maximum amplitudes during the entire monitoring period, *P* is related to the period *T* of the seasonal cycle, the latter equal to *2π/Β*, and *C* is related to the horizontal shift, relative to the beginning of the seasonal cycle. For the fitting of the data, the Levenberg–Marquardt algorithm is used to account for the nonlinearity of the least squares problem, with the results presented in Equation (1) and Figure 12. The sample is small, with data collected from only two complete seasons, and the nature of the crack (dormant or active) cannot be justified yet. Hence, the fitting will be updated once data from more seasons are collected.

(1) $$\mathrm{CR}\left(t\right)=0.9695\cdot \sin\left(0.0006963\cdot t-1.366\right)+0.0002563\cdot t+1.159$$

**Figure 13:**
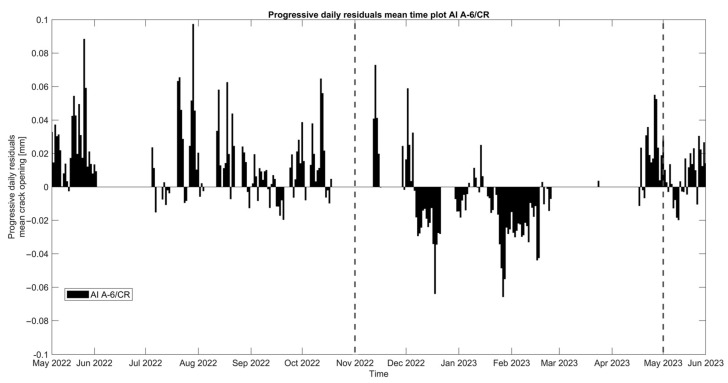
Daily evolution graph of the daily residuals of the mean crack amplitudes (J6-1-25 linear potentiometer crack-meter). Note that positive and negative residuals correspond to a daily increase and decrease in the mean crack opening, respectively.

The crack opening can be analyzed further by plotting the daily residuals of the mean daily crack amplitudes (Figure 13). The high presence of positive daily mean residuals during the first dry period (May–October 2022) is a clear indication of the progressive opening of the crack, while the vast majority of negative daily mean residuals during the first wet period (November 2022–May 2023) shows that the crack is closing.

### 3.4. Out-of-Plane Rotations

**Figure 14:**
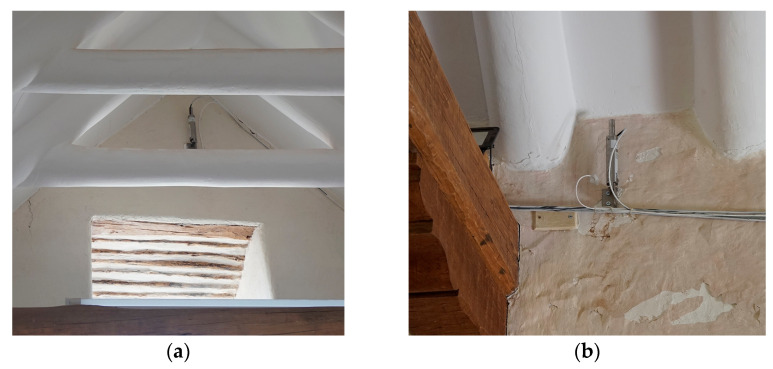
View of the MEMS tilt-meter sensors in the interior at the top of the gable end of the baptistery (**a**) and in the interior of the top eave of the north lateral wall of the nave (**b**), together with a PT100 thermocouple at each sensor location, embedded in the adobe wall.

Two highly sensitive tilt-meters are installed to monitor vertical rotations in the structure, i.e., one at the top of the gable end of the baptistery (tilt-meter 1) and one at the top eave of the north lateral wall of the nave (tilt-meter 2), shown in Figure 14. Their location is based on the findings of previous inspections and aims to investigate the cause of structural damage and potential instabilities in the northeast front of the structure, such as out-of-plane movements of the walls related to settlement actions, hygrothermal effects, and earthquakes. As for the processing of the signals of tilt-meter 1 at the top of the gable end of the baptistery (Figure 14a) and tilt-meter 2 at the top eave of the north lateral wall of the nave (Figure 14b), which were originally sampled at 20 Hz, are down-sampled to obtain a single hourly value equal to the median.

A conventional sign for the direction of angles is chosen here; namely, positive and negative angles are oriented towards the exterior and interior directions, respectively. Also, all vertical rotations are referenced with respect to the initial value, thus starting from zero degrees.

**Figure 15:**
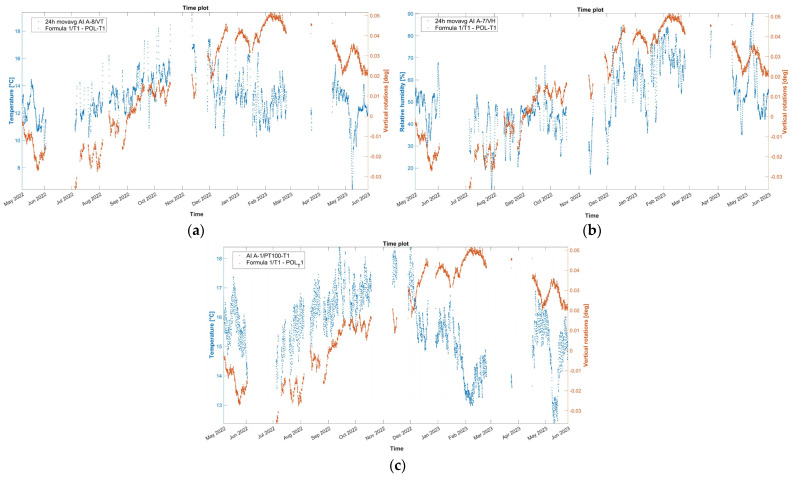
Hourly evolution graphs of vertical rotations from tilt-meter 1 at the gable end of the baptistery and (**a**) 24 h moving average of the external air temperature, (**b**) 24 h moving average of the external RH, and (**c**) wall temperature.

For tilt-meter 1, the evolution time plot is reported in Figure 15 together with the 24 h moving average of external air temperature, external RH, and internal wall temperature at the same location of the tilt-meter. In Figure 14, during the first dry season (May–October 2022), vertical rotations at the baptistery gable end progress initially along the negative axis (towards the interior). After July 2022, the trend is reversed, with a continuous increase until February 2023, with a rate of around 5.4 × 10^−3^ deg per month, reaching a maximum of around 0.05 deg. Next, from the midst of the first wet season (February 2023), the correlation with time again becomes negative, at a rate of around 8.5 × 10^−3^ deg per month and continuing until the end of the monitoring period. A strong positive correlation between angle and external environmental parameters, especially RH, emerges throughout the entire monitoring period. Instead, the correlation with the internal wall temperature (Figure 15c) is found to be complex and alternating even between seasons. In Figure 5, tilt-meter 1 sensor is able to capture the cyclic response of the gable end wall during the far distant earthquake (26 May 2022, 7:02 a.m. local Peruvian time, M7.2, 10 km of Azángaro, Peru), with a maximum magnitude of ±0.45 degrees, yet it is interpreted only as a dynamic response. Rotations are able to return to the previous state, with no residual increments recorded.

**Figure 16:**
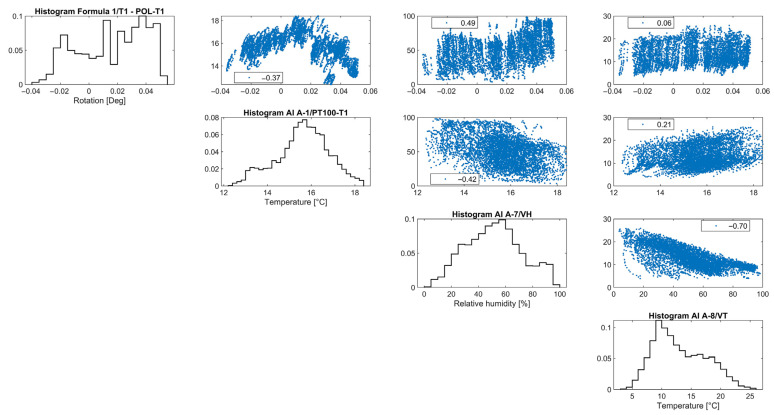
Histograms and correlation graphs of vertical rotations from tilt-meter 1 at the gable end of the baptistery for the corresponding wall temperature, the external air temperature, and RH of the monitoring period in the form of a matrix.

**Figure 17:**
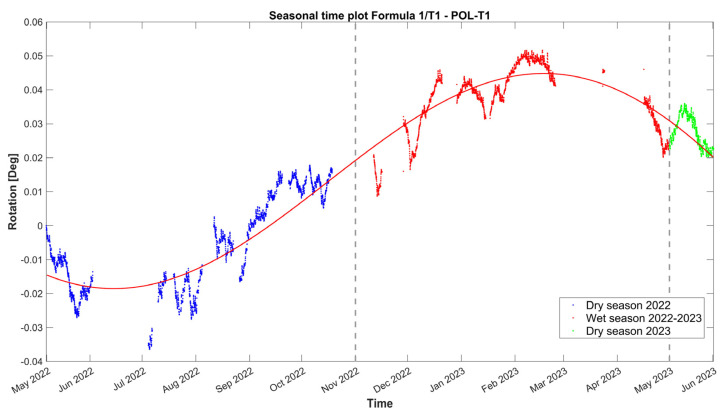
Hourly evolution graph of the vertical rotations from tilt-meter 1 at the gable end of the baptistery with a sinusoidal fit in red. Note the seasonal divisions in different colors and the *x*-axis in months. The dashed lines signify the seasonal divisions.

Overall, the evolution of vertical rotations at the gable end of the baptistery is small in terms of amplitude, and it appears to be subjected only to seasonal fluctuation. In fact, depending on the location of the rotation angle and considering the height of the gable end of the baptistery either from the interior or the exterior floor level, equal to 6.3 m and 9.5 m, respectively, the maximum recorded angle corresponds to an outward tilting of around 0.09%. Considering the correlation graphs of Figure 16, the regression function between the dataset of tilt-meter 1 and the environmental parameters appears to be poor, mostly associated with time lags, due to the thermal inertia of the adobe walls. As a first preliminary attempt, the time evolution of the vertical rotations at the gable end of the baptistery *T_1_* (*t*) is fitted by a combination of a linear trend and a sinusoidal function, as explained in Section 3.3 and [18], given in Equation (2) and shown in Figure 17.

(2) $$T_{1}\left(t\right)=-0.04322\cdot \sin\left(0.0004656\cdot t+1.323\right)-3{.678\;\times \;10}^{-6}\cdot t+0.02771$$

**Figure 18:**
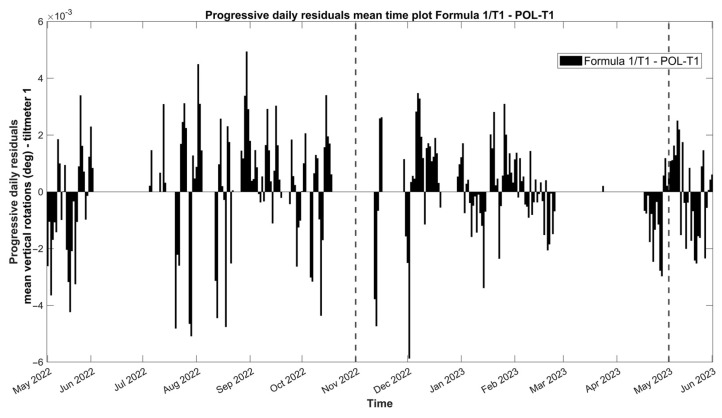
Daily evolution graph of the daily residuals of the mean vertical rotations from tilt-meter 1 at the gable end of the baptistery. Note that positive and negative residuals correspond to a daily increase and decrease in the mean crack opening, respectively. The dashed lines signify the seasonal divisions.

The positive mean daily amplitudes of vertical rotations for tilt-meter 1, as seen in Figure 18, appear to govern the part of the first wet period (July–December 2022), while several positive daily means appear to be compensated over time during the second half of the first wet period (February–May 2023).

**Figure 19:**
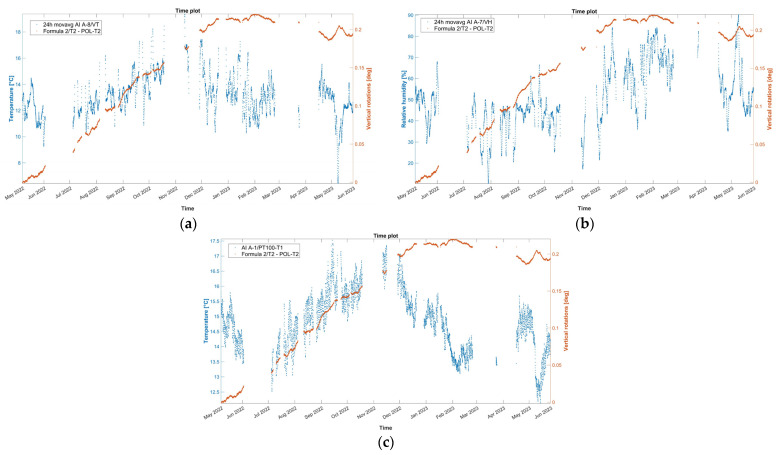
Hourly evolution graphs of vertical rotations from tilt-meter 2 on the top eave of the north nave wall and (**a**) 24 h moving average of the external air temperature, (**b**) 24 h moving average of the external RH, and (**c**) wall temperature.

Subsequently, the time evolution of the vertical rotation in degrees of the top eave of the north lateral wall of the nave, as measured by tilt-meter 2, is presented in Figure 19, together with the 24 h moving average of external air temperature, external RH, and internal wall temperature at the same location of the tilt-meter. Here, during the entire first dry season (May–November 2022) and a part of the first wet season (November 2022–January 2023), vertical rotations in the northeast part of the nave progress outwards, following a somewhat linear trend, namely around 3.0 × 10^−2^ deg per month and reaching a maximum around 0.21 degrees. Considering the height of the location of tilt-meter 2, equal to 6.85 m and 8.35 from the interior and exterior floor level, respectively, the maximum recorded vertical rotation corresponds to an outward tilting of around 0.37%. After January 2023, vertical rotations appear to stabilize, with a small negative trend, until the end of the monitoring period (June 2023). In addition, regarding the dynamic response of the wall during the captured far distant earthquake on 26 May 2022, at 7:02 a.m. local Peruvian time (M7.2, 10 km of Azángaro, Peru), dynamic rotations were registered equal to +1.50 and −1.75 degrees. Right after, vertical angles were able to return to the previous state, with no residual rotation recorded.

Similar to tilt-meter 1, the vertical rotations of tilt-meter 2 present positive correlations with the external air temperature and external RH (Figure 19a,b), while the correlation with the internal wall temperature, as seen in Figure 19c, appears to be alternating even in between seasons. Nonetheless, for this tilt-meter, a residual outward rotation remains at the end of the first year of monitoring, potentially indicating an active destabilizing phenomenon.

**Figure 20:**
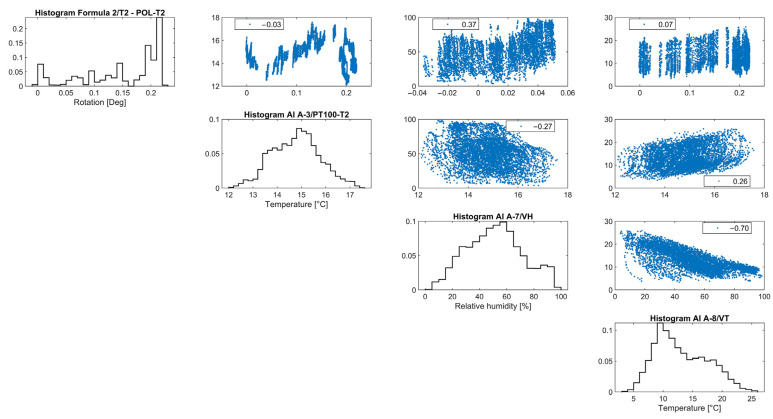
Histograms and correlation graphs of vertical rotations from tilt-meter 2, on the top eave of the north nave wall, the corresponding wall temperature, the external air temperature, and RH of the entire monitoring period in the form of a matrix.

**Figure 21:**
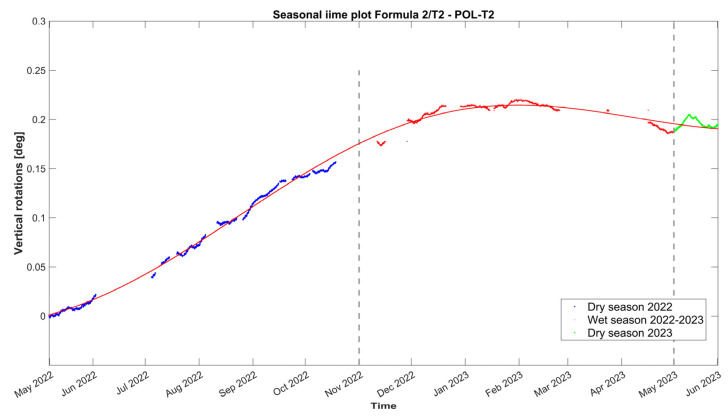
Hourly evolution graph of the vertical rotations from tilt-meter 2, on the top eave of the north nave wall, with a sinusoidal fit in red. Note the seasonal divisions in different colors and the *x*-axis in months. The dashed lines signify the seasonal divisions.

Similar to tilt-meter 1, the vertical rotations of tilt-meter 2 shown in the correlation graphs in Figure 20 present positive correlations with the external air temperature and external RH, while the correlation with the internal wall temperature appears to be alternating even in between seasons, perhaps following a polynomial function. Hence, preliminary fitting over time was attempted for the dataset of tilt-meter 2, here *T_2_*(*t*), with a combination of a linear trend and a sinusoidal function, as explained in Section 3.3 and [18], given in Equation (3) and shown in Figure 21. The progression over time of vertical rotations from tilt-meter 2 offers some indications of a progressive out-of-plane action, followed by perhaps a new state of static equilibrium in the last parts of the dataset.

(3) $$T_{2}\left(t\right)=-0.05301\cdot \sin\left(0.0005769\cdot t+1.627\right)+1{.846\;\times \;10}^{-5}\cdot t+0.05335$$

**Figure 22:**
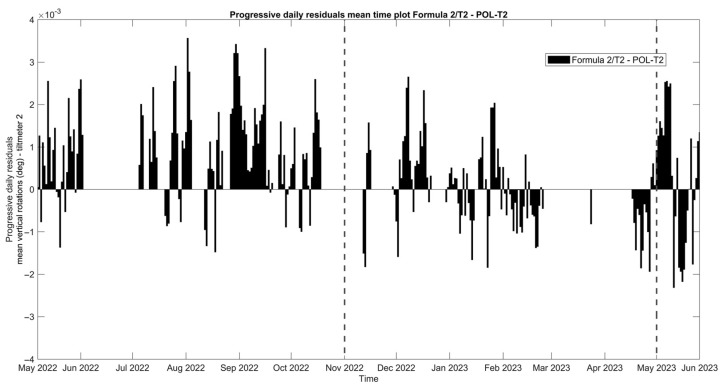
Daily evolution graph of the daily residuals of the mean vertical rotations from tilt-meter 2 on the top eave of the north nave wall. Note that positive and negative residuals correspond to a daily increase and decrease in the mean crack opening, respectively. The dashed lines signify the seasonal divisions.

From the daily mean residuals of tilt-meter 2 in Figure 22, the first dry period (May–November 2022) presents a consecutive accumulation of positive daily residuals, while only in the second part of the first wet season (February–May 2023) do they appear to be negative.

### 3.5. Modal Properties

#### 3.5.1. Ambient Vibration Testing before SHM and Mode Selection

**Figure 23:**
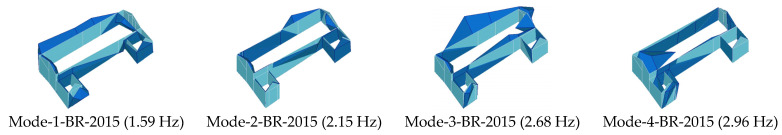
Dynamic identification tests in the un-retrofitted church of Kuñotambo (May 2015): Mode shapes obtained from the SSI-UPCX method, for the four identified modes [10].

Previous AVT defined the reference values for modal properties in the church of Kuñotambo. Initially, an AVT conducted by UMinho in May 2015 reported on the modal properties of the un-retrofitted state, here assigned the acronym BR-2015 (before retrofitting and the year of execution) [10]. This allowed for a characterization of the weak connectivity at the corners of the central nave and the inactivity of the tie beams. The first four modes, which were estimated using the Stochastic Subspace Identification Technique with Uncertainty Estimation (SSI-UPCX) method in Artemis 7.0 software [25,26], had frequencies in the range of 1.59 Hz and 2.96 Hz. The first two modes (Mode-1-BR-2015 and Mode-2-BR-2015) had complex shapes, characterized by the out-of-phase bending of the nave and presbytery, due to the infectivity of the tie beams. Mode-3-BR-2015 involved the in-phase out-of-plane bending of the nave walls, but of different amplitudes, while Mode-4-BR-2015 described the out-of-plane rotation of the east façade (Figure 23).

**Figure 24:**
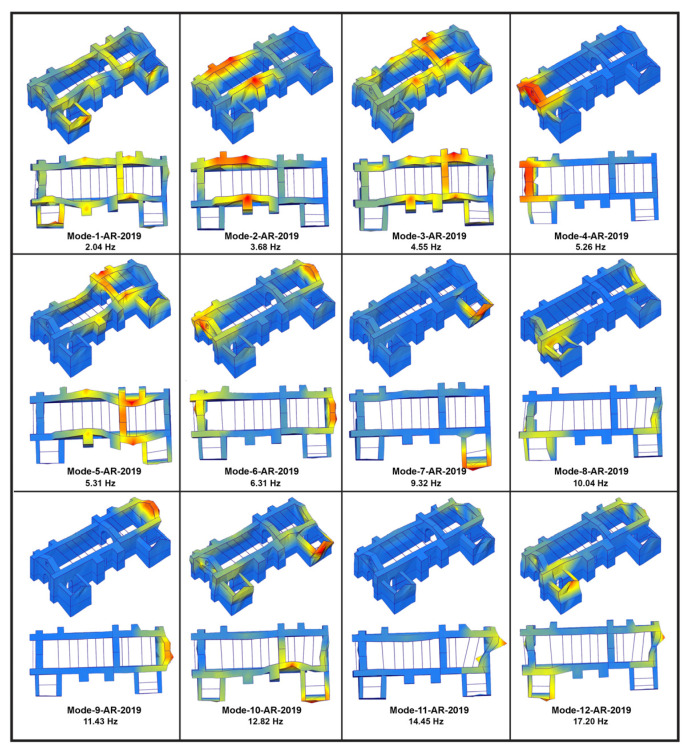
Dynamic identification tests in the retrofitted church of Kuñotambo (December 2019): Mode shapes obtained from the SSI-UPCX method for the twelve identified modes [10].

Later, during the December 2019 field campaign, UMinho conducted AVT in the retrofitted church of Kuñotambo, here assigned with the acronym AR-2019 (after retrofitting and the year of execution) [12]. The first twelve modes are presented in Figure 24. Here, the frequencies range between 2.04 Hz and 17.20 Hz. Mode-1-AR-2019 (2.04 Hz) has a complex out-of-plane bending with a third-order curvature, which mostly involves the nave and presbytery, but also a low-energy peak in the power spectral density estimate matrix, perhaps associated with the flexible roof, and thus of low importance for the global response. The retrofitted structure presents in-phase bending modes with a single curvature, namely Mode-2-AR-2019 (3.68 Hz) and Mode-3-AR-2019 (4.55 Hz). The additional stiffness from the quincha triumphal arch, the asymmetric location of the new buttresses in the south façade, the higher free height of the lateral walls of the nave, and the elevated ground floor leading to the altar appear to divide the out-of-plane modes of the nave. The walls of the presbytery appear to be stiffer than the ones of the nave, excited at a higher frequency, i.e., 4.55 Hz in Mode-3-AR-2019. Mode-4-AR-2019 (5.26 Hz) involves the out-of-plane deflection of the east façade, with a single curvature. Mode-5-AR-2019 (5.31 Hz) corresponds to an in-phase out-of-plane bending mode of the nave and presbytery walls, with a second-order curvature. Both Mode-4-AR-2019 and Mode-5-AR-2019 are closely spaced in the frequency domain, and therefore it would be difficult to distinguish them in an automated operational modal analysis (OMA). Mode-6-AR-2019 at 6.31 Hz involves the out-of-plane and out-of-phase deflection of the east and west main façades. Mode-7-AR-2019 provides the out-of-plane bending mode of the gable wall of the sacristy, at a frequency of 9.32 Hz. Mode-8-AR-2019 (10.04 Hz) is the only identified in-plane mode of the north lateral wall of the nave and presbytery, and Mode-9-AR-2019 (11.43 Hz) involves the out-of-plane bending of the west façade. Lastly, Mode-10-AR-2019, Mode-11-AR-2019M, and Mode-12-AR-2019 are local.

The retrofitted structure presents a more uniform and global behavior, compared to the AVT of the un-retrofitted state. The connectivity in the corners, the anchoring of the tie beams, the bond beam, and timber keys, both orthogonal and horizontal, resulted in higher frequencies and in-phase mode shapes, which is a direct indication of higher stiffness, enhanced connectivity, and in general, an integral (or global) structural response. As seen clearly in Figure 23 and Figure 24, the previous complex out-of-phase bending modes of the nave and presbytery, due to the infectivity of the tie beams, Mode-1-BR-2015 (1.59 Hz) and Mode-2-BR-2015 (2.15 Hz) in the retrofitted structure are replaced with two in-phase bending modes with a single curvature, Mode-2-AR-2019 (3.68 Hz) and Mode-3-AR-2019 (4.55 Hz).

**Figure 25:**
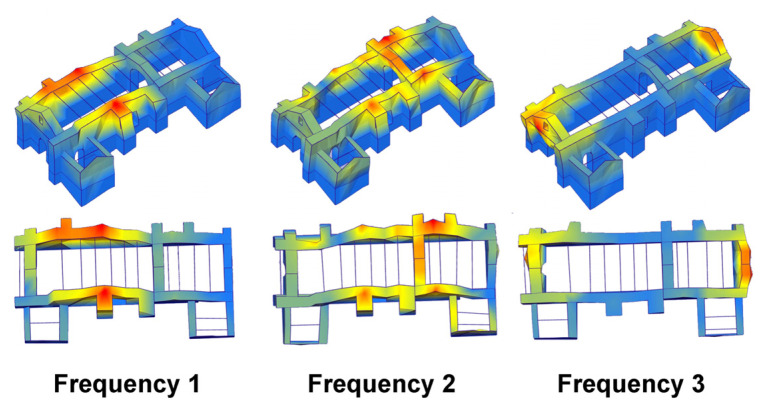
Mode shapes of the three selected modes for the SHM of the retrofitted church of Kuñotambo, as configured with the SSI-UPCX method in Artemis 7.0 software and from the AVT during the December 2019 field campaign.

Considering the automated process of AVT for the processing of the SHM monitoring data, three relevant modes of the retrofitted church are selected and monitored, namely, Mode-2-AR-2019 (3.68 Hz) and Mode-3-AR-2019 (4.55 Hz), which mostly involve the out-of-plane response of the nave and presbytery north and south lateral walls, and Mode-6-AR-2019 (6.31 Hz), which mostly activates the out-of-plane deflection of the west façade, as shown in Figure 25. These three natural frequencies are considered from this point forward as Frequency 1, 2 and 3, respectively. Here is noted that the focus on the SHM of dynamic properties is set on the global modes of the nave and presbytery, which is associated to the out-of-plane response during seismic actions. The reintegration of connections and enhancement of out-of-plane bending stiffness, mainly by means of the embedded timber bracing elements, clearly reflected on the respective natural vibration modes. Hence, monitoring of their time evolution is a clear performance indication of the retrofitting scheme and of damage identification after earthquake actions.

#### 3.5.2. SHM of Dynamic Properties

**Figure 26:**
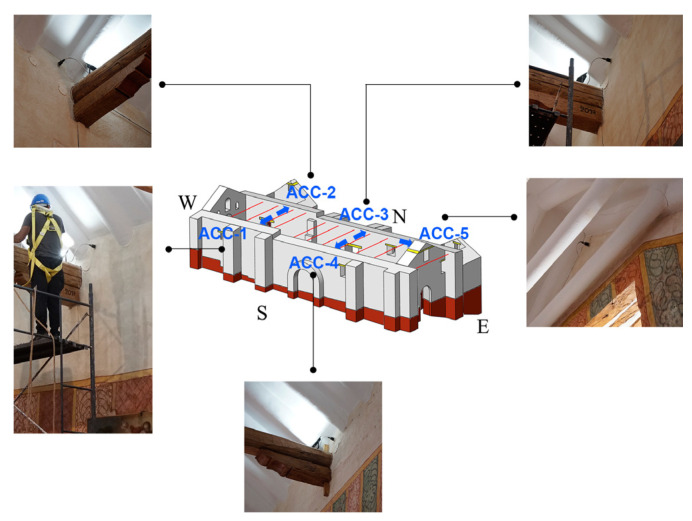
Ambient vibration testing, part of the SHM system in the retrofitted church of Kuñotambo, implemented in May 2022: placement of accelerometers at the nave and front end.

In May 2022, the continuous SHM system was installed, gathering ambient vibration data in hourly acquisitions. The setup configuration of the AVT for the SHM is based on the prior configuration of the AVT of BR-2015 and AR-2019, with the location and direction of the five uniaxial accelerometers shown in Figure 26. Here, the objective for the dynamic monitoring of the structure is to extract the selected dynamic properties that are indicative of the out-of-plane response of the north, south and east façade of the structure and monitor their evolution over time to identify progressive damage, especially after an earthquake. In total, and accounting for power interruptions, around 5500 events were recorded and processed, in the monitoring period of around 12 months.

For the processing, an automatic frequency domain decomposition (FDD) method was used, as described in [25,26], which is considered a well-established and highly accurate method for the estimation of the modal properties of a monitored system. The mode identification automated process entails two steps. First, the power spectral density (PSD) matrix is estimated. Second, accounting for windows of lower and upper boundaries, set for each mode, an automatic pick-peaking algorithm checks the modes around the peak and the point with the highest modal assurance criterion (MAC) is chosen. In the present processing stage, and besides the detrending of the raw acceleration signals, no other digital processing was performed, i.e., filtering, resampling etc. Yet, considering the quality of each hourly acquisition, some specific signals were found noisier than others, which resulted in a higher dispersion of the natural frequencies, and thus, additional digital processing will be considered in the future. Here, a high-frequency resolution of the PSD estimate was chosen, equal to 20,480 data points. As an output, a single modal identification set, i.e., of natural frequencies and mode shapes, is produced per hour.

**Figure 27:**
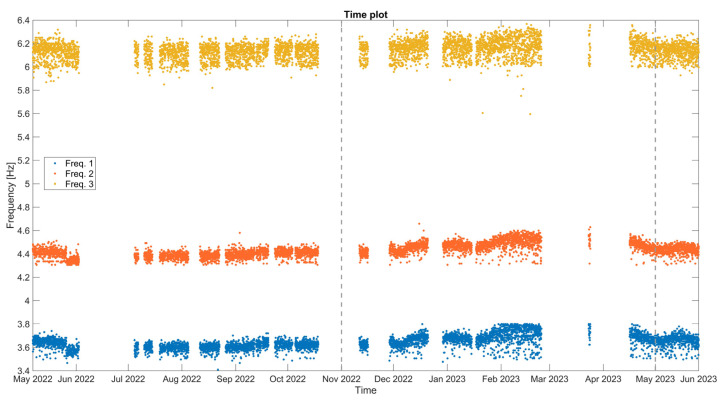
Hourly time evolution of the three selected natural frequencies of the structure. The dashed lines signify the seasonal divisions.

The time plot of the hourly set of the three chosen natural frequencies (Frequency 1, 2, and 3) is presented in Figure 27 and a set of basic statistics for the entire monitoring period and each season, i.e., first dry season (May–October 2022), first wet season (November 2022–May 2023) and second dry season (May 2023), are reported in Table 4. The first mode (mean frequency equal to 3.64 Hz) is an in-phase bending mode of single curvature involving mostly the lateral walls of the nave, whereas the second mode (mean frequency equal to 4.42 Hz) is similar, also involving the lateral walls of the presbytery. The third mode (mean frequency equal to 6.15 Hz) activates the east façade and the walls of the presbytery in out-of-plane in-phase deflections. These modes are also identified during the AVT of the December 2019 field campaign, with minor differences in the natural frequencies.

From the time evolution plot of Figure 27, all three natural frequencies are fairly stable, but with small and alternating trends, either positive or negative, which appear to be attributed to seasonal cycles. Frequencies 1, 2, and 3 range between 3.4 and 3.8 Hz, 4.3 and 4.7 Hz, and 5.6 and 6.4 Hz, respectively, with low coefficients of variation between 1 and 2% (Table 4). Considering the median and mean values, those are found to be almost identical in all three natural frequencies, which indicates that the data have a symmetrical distribution. In fact, except for extreme earthquake events, the detection of damage from the time evolution of natural frequencies is based on time trends and small changes. Considering the natural frequencies in each season, as seen in Table 4, the mean and median values of the three natural frequencies present a slight increase during the wet season 1 (November 2022–May 2023), which needs confirmation with more data but may be due to foundation soil swelling.

**Figure 28:**
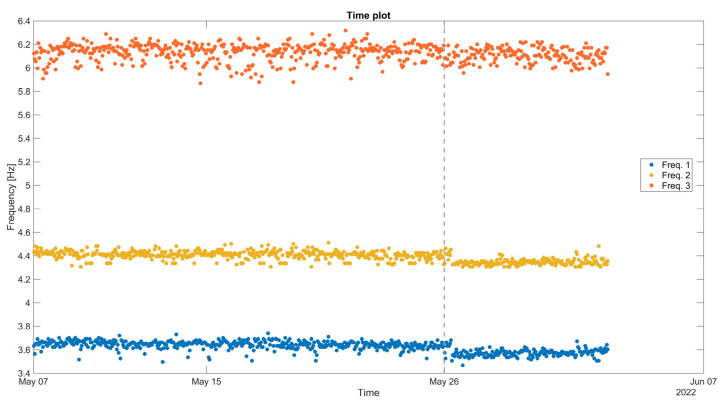
Window of hourly time plot for the three selected natural frequencies from May to June 2022. Note the time division of 26 May 2022, where the earthquake event occurred, at 7:02 am local Peruvian time, with an M7.2 magnitude and an epicenter near Puno, 10 km from Azángaro, Peru.

Focusing on a part of the first dry season (May–June 2022) and on 26 May 2022, during the event of a magnitude M7.2 earthquake, 200 km from the structure, and with a recorded maximum horizontal base acceleration of 0.012 g, the monitored natural frequencies of the structure are affected, exhibiting a sudden drop right after the event (Figure 28). The drop is larger for the lower Frequencies 1 and 2, around 0.1–0.2 Hz, and is a clear indication of stiffness loss and damage.

**Figure 29:**
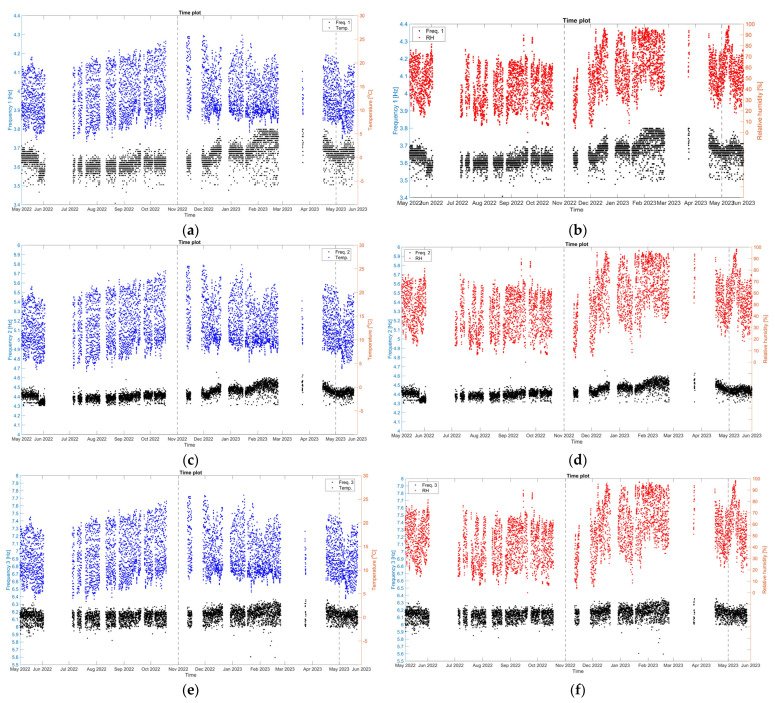
Hourly time evolution of the natural frequencies: Frequency 1 in correlation with the external air temperature (**a**) and the external RH (**b**); Frequency 2 in correlation with the external air temperature (**c**) and the external RH (**d**); and Frequency 3 in correlation with the external air temperature (**e**) and the external RH (**f**). The dashed lines signify the seasonal divisions.

Considering the correlation of the natural frequencies of the structure with the environmental parameters, the time plots of the three monitored natural frequencies, with the external air temperature and RH are presented in Figure 29. Here, all three monitoring frequencies present the same seasonal variation, mostly under a positive correlation with the external RH, while the type of correlation with the external temperature is found to be difficult to identify. During the first dry season (May–October 2022), and besides the drop after the earthquake of 26 May 2022, the three monitored natural frequencies are found rather stable, which can be attributed to the relatively stable range of the external RH values, even though the external temperature has a positive trend. Next, in the first wet season (November 2022–May 2023), the increase in external RH is in correlation with the positive trend in the natural frequencies, whereas the drop of the external RH, after the middle of February 2023 until June 2023 is followed by the negative trend in natural frequencies. Such an observation is also consistent with [24]. Yet, the amount of processed data is considered insufficient and more seasons are needed to establish a correlation fit and calculate time delays within natural frequencies and environmental parameters.

## 4. Discussion

A permanent structural health monitoring (SHM) system was designed and implemented in the church of Kuñotambo to continuously observe structural parameters, such as the out-of-plane rotations of walls, crack opening, temperature, relative humidity, modal properties, and strong ground motions. The system, consisting of various sensors and devices, has been operational since May 2022, and the first analysis of the recorded data between May 2022 and May 2023 is presented. The initial data processing yielded several significant findings that contribute to the broader understanding of structural health monitoring (SHM) in historic earthen buildings, also highlighting the profound impact of environmental conditions on the interpretation of the structural integrity of the church. Yet, accounting for damage identification and regression models in time plots of crack width, rotations, and dynamic properties, longer monitoring periods are needed.

Environmental conditions in Kuñotambo are characterized by a drier and colder season from May to October and a wet and rainy season from November to April. External air temperature and relative humidity data were recorded using various sensors. Internal air temperature and relative humidity were found to be relatively stable and less variable than those of the exterior due to the thermal inertia of the thick adobe walls. The wet season showed higher internal RH values compared to the dry season, with an increase of 36% in median values, while the internal air temperature during the wet season was also higher, with a 15% increase in median values (these values are normalized by the dry season). The wall temperature sensors placed inside the church showed consistent median values and less variation compared to external temperatures. Also, the internal wall temperatures exhibited a time lag compared to external environmental parameters due to the thermal inertia of the thick adobe walls. The time lag between internal wall temperatures and external air temperature/RH was around 4–6 h. The data collected revealed important correlations between environmental parameters and structural responses, with static and dynamic data influenced by hygrothermal cycles, showing seasonal fluctuations, with hysteretic and nonlinear relationships, attributed to time lags due to the thermal inertia of adobe masonry. Hence, correlations through simple linear models are inadequate, pointing to the need for more sophisticated multivariate analyses, based on long monitoring periods, to capture the complexity of these interactions.

The continuous monitoring of a crack in the staircase void with an LVDT crack-meter revealed seasonal fluctuations in the crack width due to the hygrothermal cycles of adobe masonry, highlighting the need for extended monitoring to determine the nature and evolution of the crack. A minor-intensity earthquake was noted to cause a non-recoverable crack width increase of 0.14 mm. The correlations between crack openings and environmental parameters, such as external air temperature, relative humidity, and internal wall temperature, appeared to be hysteretic and complex, making linear regression models inappropriate. Thus, further data collection and multivariate analysis were needed to establish robust correlations and time lag compensations. Anticipating data collection from two complete seasonal cycles, the rate of active crack progression, possibly due to movements at the foundation or foundation soil, can be established, leading to informed decisions for strengthening.

Two highly sensitive tilt-meters monitored vertical rotations at specific locations in the structure. Additionally, thermocouples were used to measure wall temperatures, aiming at evaluating the temperature effects. The data collected from the tilt-meters and thermocouples were processed and the results showed a sudden increase during earthquakes, which fully recovered. Moreover, for the tilt-meter located at the gable end of the baptistery, vertical rotations exhibited a seasonal fluctuation, while their progression was small and alternating in direction. The correlation with environmental parameters, such as external relative humidity (RH) and temperature, showed more consistency compared to the internal wall temperature. For the tilt-meter located at the top eave of the north lateral wall of the nave, vertical rotations progressed outwards during the first dry season and then stabilized in the wet season. Here, there was a correlation between external air temperature and RH, while the correlation with the internal wall temperature was complex and alternating. In both cases, preliminary fitting attempts with linear trends and sinusoidal functions were made, but the small time sample of only two complete seasons limited the accuracy of the conclusions. More seasonal data are necessary to update the fitting.

Three natural frequencies and mode shapes were identified and monitored continuously over time, based on hourly acquisitions of five uniaxial accelerometers placed at the top of the walls in the nave and the façade, and processed using an automatic frequency domain decomposition algorithm. The natural frequencies were found to be stable, with small alternating trends due to seasonal cycles, while a likely dependence on the relative humidity was identified, reflected by a positive correlation, especially during the wet season. The M7.2 earthquake event on 26 May 2022, with its epicenter 200 km away from the structure, caused a sudden drop in the monitored natural frequencies of around 0.1–0.2 Hz, which is indicative of stiffness loss and minor damage. In future research, the monitoring of mode shapes by means of MAC values will offer additional information on damage identification.

In general, for all collected monitoring data, seasonal effects were responsible for oscillating variations, sometimes due to complex nonlinear correlations with environmental parameters. Given the two distinct wet (November–April) and dry (May–October) seasons in the region, SHM data collection of two annual cycles is considered necessary to characterize oscillations and time lags between environmental, static, and dynamic data.

In addition, retrofitted adobe structures, given their high deformability, are subjected to complex interactions and secondary deformations from drying shrinkage in new adobe structural elements, cyclic expansions and contractions of embedded timber elements, and the additional load from new roofs. Hence, despite the indications of destabilizing actions, namely settlement, and since only one complete yearly cycle has been acquired, for the moment it is not possible to firmly determine the presence of trends in the data. This highlights the critical importance of continued monitoring over multiple seasonal cycles to refine regression models and achieve a more accurate understanding of the long-term structural behavior.

In conclusion, the importance of a carefully designed and sustained SHM system and the integration of advanced analytical techniques to accurately assess and mitigate the effects of environmental, seismic, and static actions on earthen heritage buildings is highlighted. By leveraging the detailed insights gained from this SHM system, future efforts can be more effectively directed towards the conservation and protection of similar historic structures.

## Figures and Tables

**Table 1:** Statistics of ambient relative humidity, air temperature, and wall temperature for all the environmental sensors, during the entire monitoring period.

	External		Internal						Internal Walls		
			Altar Niche		Arch Nave		Sotocoro				
	Temp/RH PT100		Data Logger						PT100 T1	PT100 CR	PT100 T2
	Air Temperature (°C)	Relative Humidity (%)	Air Temperature (°C)	Relative Humidity (%)	Air Temperature (°C)	Relative Humidity (%)	Air Temperature (°C)	Relative Humidity (%)	Wall Temperature (°C)		
Median	12.29	51.43	14.00	50.00	14.00	53.00	14.30	47.10	15.69	15.21	14.82
Mean	13.11	51.43	13.87	50.69	13.99	53.40	14.41	48.26	15.61	15.17	14.78
Std	4.41	20.48	1.32	10.82	1.07	12.42	1.21	9.85	1.20	0.86	0.98
Min	3.38	3.71	10.5	19.0	10.50	15.00	11.6	21.4	12.37	12.88	12.12
Max	25.86	98.23	18.0	73.0	18.76	77.00	18.2	69.0	18.40	17.53	17.57
1st Quart.	9.67	36.20	13.00	43.00	13.32	43.50	13.55	41.10	14.92	14.52	14.05
3rd Quart.	16.49	64.96	15.00	60.00	14.50	64.50	15.20	56.90	16.43	15.81	15.43

**Table 2:** Statistics of ambient relative humidity, air temperature and wall temperature for all the environmental sensors, during the dry season period of May to October 2022 (season 1).

	External		Internal						Internal Walls		
			Altar Niche		Arch Nave		Sotocoro				
	Temp/RH PT100		Data Logger						PT100 T1	PT100 CR	PT100 T2
	Air Temperature (°C)	Relative Humidity (%)	Air Temperature (°C)	Relative Humidity (%)	Air Temperature (°C)	Relative Humidity (%)	Air Temperature (°C)	Relative Humidity (%)	Wall Temperature (°C)		
Median	12.41	43.24	13.50	45.00	14.09	41.70	14.32	42.20	16.21	15.46	15.05
Mean	13.09	42.46	13.47	43.88	14.16	40.90	14.39	41.48	16.16	15.42	15.03
Std	4.64	16.87	1.33	7.33	1.33	6.68	1.29	6.13	0.86	0.72	0.88
Min	3.38	6.47	10.5	19.0	11.0	20.7	11.6	21.4	13.56	13.14	12.52
Max	24.58	89.69	17.5	64.0	18.1	59.2	18.0	59.0	18.38	17.11	17.57
1st Quart.	9.40	28.25	12.50	39.50	13.20	37.40	13.40	38.40	15.58	14.91	14.37
3rd Quart.	16.95	56.33	14.50	49.00	15.10	45.20	15.31	45.33	16.75	15.97	15.65

**Table 3:** Statistics of ambient relative humidity, air temperature and wall temperature for all the environmental sensors, during the wet season of November 2022 to April 2023 (season 2).

	External		Internal						Internal Walls		
			Altar Niche		Arch Nave		Sotocoro				
	Temp/RH PT100		Data Logger						PT100 T1	PT100 CR	PT100 T2
	Air Temperature (°C)	Relative Humidity (%)	Air Temperature (°C)	Relative Humidity (%)	Air Temperature (°C)	Relative Humidity (%)	Air Temperature (°C)	Relative Humidity (%)	Wall Temperature (°C)		
Median	12.47	59.47	14.50	60.00	14.37	58.00	14.50	57.20	15.35	15.13	14.81
Mean	13.54	59.69	14.51	56.98	14.53	55.15	14.66	54.65	15.28	15.14	14.79
Std	4.21	20.77	1.07	10.10	1.09	9.58	1.09	8.77	1.24	0.87	0.95
Min	5.37	3.71	12.0	20.5	12.1	23.0	12.5	25.8	12.99	13.63	13.12
Max	25.86	97.15	18.0	73.0	18.8	70.7	18.2	69.0	18.40	17.53	17.34
1st Quart.	10.08	44.74	13.50	50.50	13.72	48.90	13.84	48.90	14.32	14.31	13.95
3rd Quart.	16.62	76.53	15.00	65.00	15.19	62.70	15.28	61.70	16.01	15.78	15.32

**Table 4:** Statistics of the three selected natural frequencies as processed over time.

	Freq. 1 [Hz]				Freq. 2 [Hz]				Freq. 3 [Hz]			
	Total	Season 1	Season 2	Season 3	Total	Season 1	Season 2	Season 3	Total	Season 1	Season 2	Season 3
Median	3.64	3.61	3.68	3.66	4.42	4.39	4.47	4.44	6.15	6.13	6.17	6.14
Mean	3.64	3.61	3.68	3.66	4.43	4.39	4.47	4.44	6.14	6.12	6.17	6.13
St. dev.	0.06	0.04	0.06	0.04	0.06	0.04	0.05	0.03	0.07	0.07	0.08	0.06
COV	1.6%	1.1%	1.6%	1.1%	1.4%	1%	1.1%	1.0%	1.1%	1.1%	1.3%	1.0%
Min	3.41	3.41	3.48	3.50	4.31	4.31	4.31	4.31	5.60	5.82	5.60	5.93
Max	3.80	3.74	3.80	3.78	4.66	4.58	4.66	4.51	6.40	6.32	6.37	6.30
1st Quart	3.60	3.59	3.64	3.64	4.39	4.38	4.44	4.41	6.09	6.07	6.12	6.09
3rd Quart	3.68	3.63	3.71	3.68	4.46	4.41	4.50	4.46	6.19	6.17	6.22	6.18

## Data Availability

Data will be available upon a reasonable request to the authors.
